# Synthesis and liquid crystalline properties of new triazine-based π-conjugated macromolecules with chiral side groups

**DOI:** 10.3906/kim-1912-51

**Published:** 2020-06-01

**Authors:** Nihat AKKURT, Mohammed Hadi Ali AL-JUMAILI, Hale OCAK, Fatih ÇAKAR, Lokman TORUN

**Affiliations:** 1 Chemistry Department, Faculty of Science and Art, Yildiz Technical University , Istanbul Turkey; 2 Chemistry Department, Faculty of Science and Art, Kirklareli University, Kirklareli Turkey; 3 TORKIM R&D Chemical Industry, YTÜ Technopark Incubation Centre, Istanbul Turkey

**Keywords:** Triazine, ionic liquid crystals, hydrogen bonding, star-shaped triazine, Sonogashira coupling

## Abstract

In this study, we reported the synthesis of a new tribranched macromolecule liquid crystal with triazine in the centre. The central triazine core is bonded via sequences of Sonigashira coupling to 3 triazine unites through acetylenic bridges. The triazines at the periphery are substituted with 2 chiral citronellyloxy side groups. The salt of the resulting star-shaped macromolecule, which was oily at room temperature, with 4-dodecyloxybenzoic acid at 1:1 ratio exhibited a Smectic C (SmC) mesophase. The liquid crystalline properties of organic salt were investigated using DSC (differential scanning calorimetry) and POM (polarizing optical microscopy).

## 1. Introduction

Liquid crystals possessing properties of both liquids and solids have been focus of intensive research activities from industrial and academic points of views due to their technological importance and wide commercial application in last few decades. Their widespread use includes in consumer electronic, office equipment, calculators, watches, stereos, etc. Perhaps the most important applications are found on liquid crystal displays (LCDs), which have a dominant position in consumer electronics [1-6].

There is a growing interest in the synthesis and investigation of nonconventional liquid crystals to discover new LC phases and low-temperature applications, which have been regarded as new models in the progression of LC science and technology as they are capable of exhibiting unique physical properties and uncommon phase transitions [1,3,4,6].

In the studies on liquid crystal materials, intensive efforts have been devoted to the synthesis of liquid crystals with star-shaped topologies to investigate their potential applications, as they may possess unique properties. Many examples of such liquid crystalline compounds, which include benzene and triazine cores and alkyl chains in the periphery, exhibit interesting LC phases [7-15].

Ionic liquid crystals can be considered as the materials that combine the properties of liquid crystals and ionic liquids. A growing interest has been devoted to the research activities in the field of ionic liquids worldwide [16-18]. One of the important properties of ionic liquids is that these compounds have very low vapor pressures and can substitute volatile organics. Other applications include the use of solvents and batteries for extraction processes, as an electrolyte for fuel cells and dye-sensitive solar cells [18]. A large number of literature reports are available on the liquid crystalline properties of the charged materials containing anions and cations [15,19,20].

One approach to obtain ionic liquids is by converting an organic material into its salt. This can be made by mixing the organic substance, which has hydrogen bond accepting heteroatoms, with a benzoic acid derivative with long alkyl chain in appropriate ratios. This is a useful and practical way of converting nonliquid crystal materials into materials with mesomorphic properties [21,22].

In this study, we reported the synthesis and the LC properties of a triarmed organic material utilizing 1,3,5-triazine units in the core and on the periphery, which were substituted with chiral citronellyloxy groups. The 1:1 mixture of this compound with 4-dodecyloxybenzoic acid presented a liquid crystal material, which was characterized by FTIR, ^1^H NMR, and ^13^C NMR analyses. The mesomorphic properties were investigated using POM and DSC.

## 2. Synthesis and characterization

**Scheme 1:**
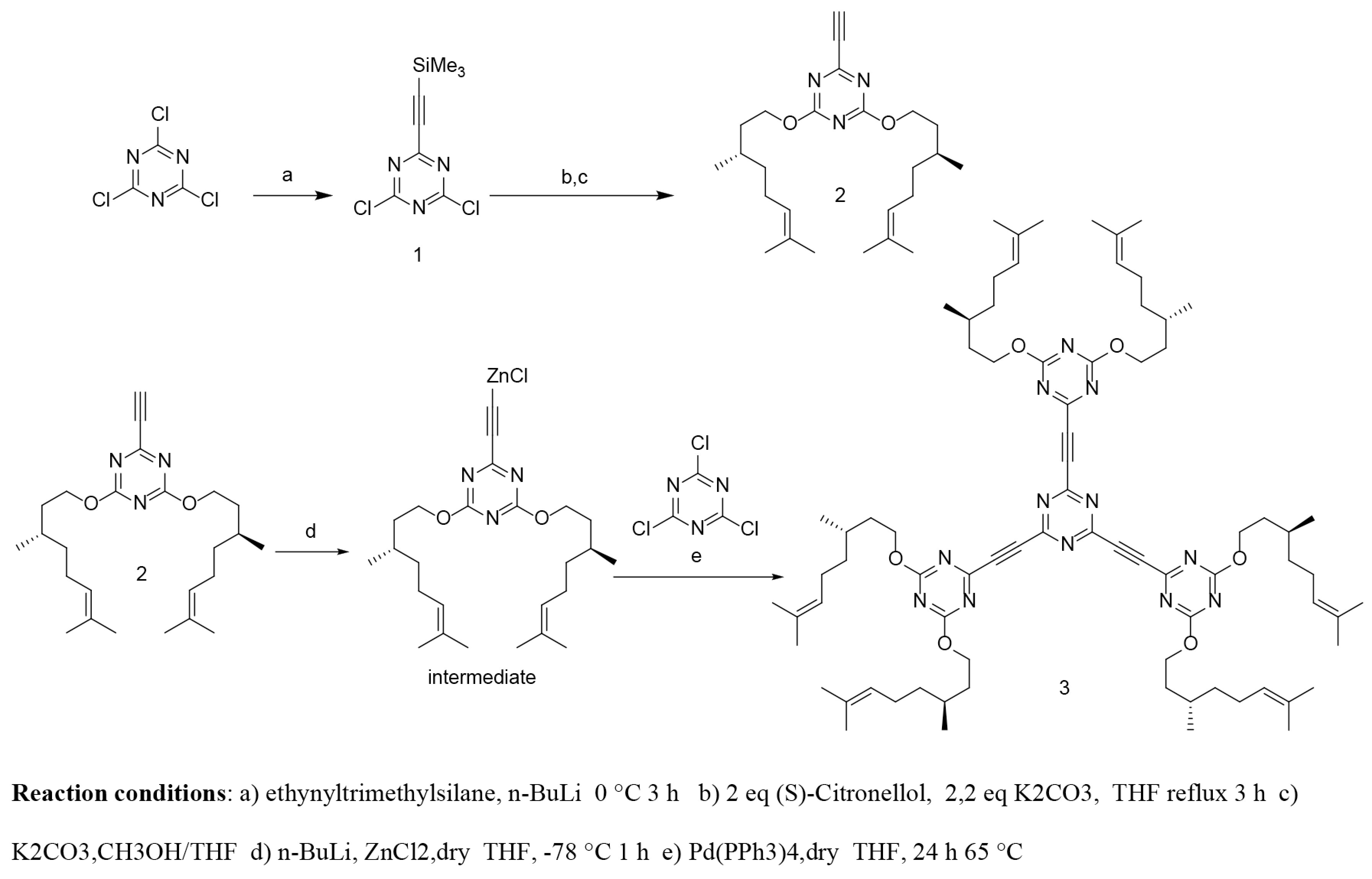
Synthesis of 2,4,6-tris((4,6-bis((S)-citronellyloxy)-1,3,5-triazin-2-yl)ethynyl)-1,3,5-triazine (3).

Synthesis of the triarmed macromolecule with chiral citronellyloxy side groups is outlined in Scheme 1 (Synthesis of 2,4,6-tris((4,6-bis((S)-citronellyloxy)-1,3,5-triazin-2-yl)ethynyl)-1,3,5-triazine (3)).

The TMS protected acetylene was treated with 1.3.5-tricloro-2,4,6-triaizen and subsequently with citronellyloxy groups followed by desililylation afforded intermediate compound 2 (Scheme 1). The HRMS analysis showed m/z [M+H]^+^ calculated for C_25_H_39_N_3_O_2_ : 414,304; found 414.312 for of compound 2. H NMR and CNMR data for compound 2 are provided in the experimental section (See supporting information).

**Scheme 2:**
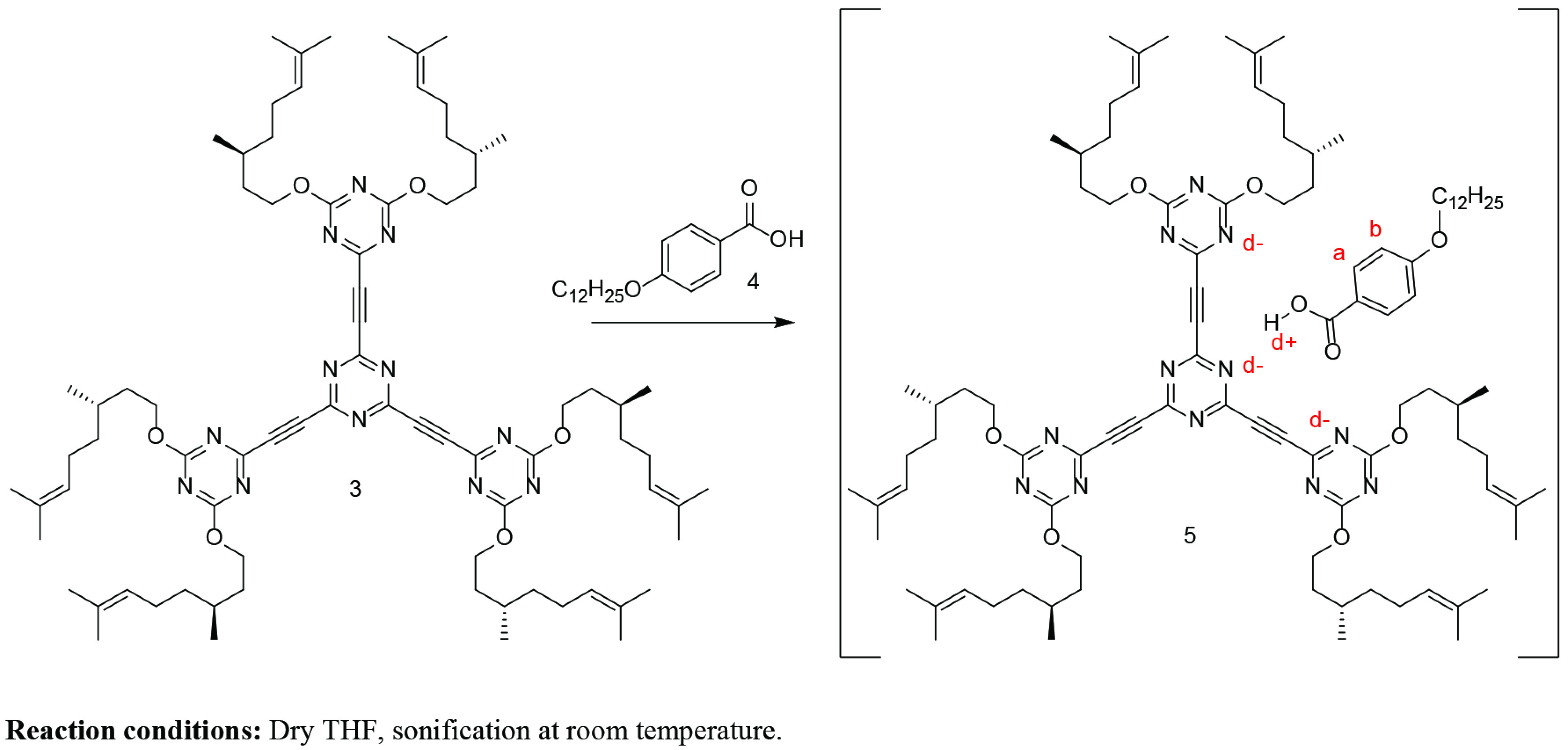
Synthesis of organic salt 5.

A Negishi protocol was applied for the synthesis of RZnCl intermediate, which then coupled with triazine in the presence Pd catalyst to afford the targeted macromolecule 3 in 34% yield as colourless oily material. Compound 4 was synthesized according to literature [23]. Converting compound 3 into its alkoxy benzoic acid salt 5 was necessary in order to obtain a solid material at room temperature, which made it possible to obtain an observable phase transitions under POM (Scheme 2). Structural characterizations for compounds 3 and 5 were confirmed by ^1^H NMR, ^13^C NMR, HRMS, and FTIR. The HRMS analysis showed m/z [M+3Na]^3+^ calculated for (C_78_H_114_N_12_Na_3_O_6_)^3+^ : 461,289; found 461,292 for compound 3. The formation of organic salt 5 was confirmed by the shift of the carbonyl stretching frequency from 1680 cm^-1^in free acid compound 4 to 1650 cm^-1^in the salt 5. In addition, the difference between the relevant proton and carbon chemical shift in the free acid 4 and salt 5 provided other confirming interaction between acid for and compound 3, which resulted the formation of salt 5 (See supporting information).

### 2.1. Experimental section

**Synthesis of 2,4-dichloro-6-((trimethylsilyl)ethynyl)-1,3,5-triazine (1) [24]**

Trimethylsilylacetylene (1.73 g, 17.6 mmol) was dissolved in dry THF (15 mL). n-Butyllithium (11.0 ml, 17.6 mmol, 1.6 M in hexane) was then added dropwise by a syringe under argon atmosphere. The reaction mixture was stirred for ^1^H at 0 °C in an ice bath. A solution of 2,4,6-trichloro-1,3,5-triazine (3.24 g, 17.5 mmol) dissolved in dry THF (8 mL) was added to the mixture slowly and the resulting mixture was stirred for 2 h at 0 °C, and it was allowed to warm up to room temperature with stirring for additional ^1^H. The solution was poured into ethyl acetate (25 mL) and the organic layer was washed with water 2 times (2 × 25 mL). The organic layer was dried with sodium sulphate, filtered and the solvent was rotary evaporated affording compound 1 (1.43 g, 33%), which was used without purification. ^13^C NMR δ 172.4, 162.3, 107.0, 99.7, and –0.01.

**Synthesis of 2,4-bis((S)-citronellyloxy)-6-ethynyl-1,3,5-triazine (2)**

**Step 1:** A mixture of 2,4-dichloro-6((trimethylsilyl)ethynyl)-1,3,5-triazine (compound 1, 1.23 g, 5 mmol), (S)-citronellol (1.72 g, 11.0 mmol), and K_2_CO_3_ (1.67 g, 11.0 mmol) in 25 mL THF was stirred at 70 °C for 3 h. The reaction mixture was allowed to warm up to room temperature. The corresponding reaction mixture was poured into ethyl acetate (25 mL) and the organic layer was washed with water 2 times (2 ×15 mL), dried with sodium sulphate and the solvent evaporated under reduced pressure. The corresponding compound was directly used without isolation and purification in the next step.

**Step 2:** The mixture of corresponding compound 2 (2,4-bis((S)-citronellyloxy)-6-((trimethylsilyl)ethynyl)-1,3,5-triazine) (1.39 g, 3 mmol), K_2_CO_3_ (0.46 g, 3.30 mmol) in 1:1 MeOH/THF (30 mL) was stirred 24 h at room temperature. The solution was poured into ethyl acetate (25 mL) and the organic layer was washed with water (2 ×10 mL) and dried with Na2 SO4 , filtered. The solvent was evaporated under reduced pressure. The residue was purified by column chromatography with hexane/ethyl acetate (5:1) as an eluent to give yellow oily materials (2) with yield (0.65 g, 47%). ^1^H NMR (500 MHz, CDCl_3_) δ 5.16–5.04 (m, 2 H CH=C(CH_3_)_2_), 4.03 (s, 1H,HC≡C), 3.76–3.59 (m, 4 H), 2.05 –1.92 (m, 4H), 1.71–1.57 (m, 16H), 1.44–1.30 (m, 2H), 1.24 – 1.14 (m, 4H), 0.93–0.88 (m, 6H). ^13^C NMR (126 MHz, CDCl_3_) δ 173.5, 171.2, 131.2, 124.7, 84.7, 77.3, 77.0, 76.8, 70.7, 61.1, 39.8, 37.2, 31.6, 29.2, 25.7, 19.5, 17.6. HRMS = (M+H)^+^_calc_ for C_25_H_39_N_3_O_2_ = 413.30. (M+H)^+^_found_ = 414.31.

**Synthesis of 2,4,6-tris((4,6-bis (S)-citronellyloxy)ethynyl)-1,3,5-triazine (3) [25]**

n-BuLi (0.45 mL, 1.20 mmol) was added dropwise to a stirred solution of compound 2 (0.49 g, 1.20 mmol) in dry THF under argon atmosphere at –78 °C, and then anhydrous ZnCl2 (0.16 g, 1.20 mmol) in THF was dropwise added into the solution and the mixture was stirred at –78 °C for about ^1^H, and then warmed up to room temperature. A solution of cyanuric chloride (0.05 g, 0.30 mmol), Pd(PPh3)4 (0.02 g, 5% eq ) in 10 mL of THF was added dropwise and the mixture was stirred 65 °C for 24 h. The mixture was cooled to room temperature. Water (10 mL) and chloroform (10 mL) were added. The aqueous layer was extracted with chloroform (2 ×10 mL). The combined organic layers were then washed with brine, dried over Na2 SO4 , filtered and evaporated under reduced pressure. The residue was purified by column chromatography hexane/ethyl acetate (6:1) (0.04 g oily material, yield 34%). ^1^H NMR (500 MHz, CDCl_3_) δ 5.11–4.93 (m, 6H), 4.15–3.97 (m, 12H), 1.94–1.84 (m, 12H), 1.64–1.47 (m, 48H), 1.38–1.26 (m, 12H), 1.15–1.06 (m, 6H), 0.83 (dd, J = 7.8, 4.^1^Hz, 18H). ^13^C NMR (126 MHz, CDCl_3_) δ 172.5, 172.1, 170.0, 130.1, 123.7, 108.9, 102.6, 76.4, 76.1, 75.9, 61.9, 35.9, 28.4, 24.6, 24.3, 19.9, 18.3, 16.5. HRMS M^+^_calc_ for C_78_H_114_N_12_O_6_ = 1315.8 M^+^_found_ = 1315.9.

**Synthesis of 4-(dodecyloxy) benzoic acid (4-DBA) (4) [23]**

A solution of 4-hydroxy benzoic acid (16.43 mmol), 1-bromododecane (11 ml, 46 mmol, 2.8 eq), and KOH (2.58 g, 46 mmol, 2.8 eq) in ethanol (50 mL) was heated under reflux for 72 h. The resulting mixture was hydrolysed with 10% aqueous potassium hydroxide (25 mL) under refluxing overnight, after which the mixture was cooled to room temperature and acidified with HCl (6 M). The precipitate was filtered, washed with water and recrystallized from ethanol to give the pure product 4-dodecyloxybenzoic acid white solid (4), 4.55 g 91 % yield. ^1^H NMR (500 MHz, CDCl_3_) δ 8.05 (d, J = 8.8 Hz 2 Ar-H), 6.93 (d, J = 6.8 Hz 2 Ar-H), 4.01 (t, J = 6.4 Hz, 2H OCH_2_) , 1.96–1.68 (m, 2H), 1.52–1.27 (m, 18H), 0.89 (t, J = 7.0 Hz, 3H).

**Synthesis of organic salt (5)**

4-Hydroxy benzoic acid (4-DBA, 4) mesogenic unit was added into 2,4,6-tris((4,6-bis(S)-citronellyloxy-1,3,5- triazin-2-yl)ethynyl)-1,3,5-triazine (3) with one to one ratio. The resulting solution in dry THF was sonicated for 10 min at room temperature until the solution has become transparent. Then, the solvent was removed in vacuum. ^1^H NMR (500 MHz, CDCl_3_) δ 7.98 (d, J = 8.8 Hz, 2H), 6.86 (d, J = 8.9 Hz, 2H), 5.08–4.95 (m, 6H), 4.06–3.99 (m 12H), 3.95 (t, J = 6.6 Hz, 2H), 2.00–1.84 (m, 14H), 1.77–1.70 (m, 3H), 1.65–1.52 (m, 30H), 1.51–1.44 (m, 8H), 1.43–1.32 (m, 12H), 1.31–1.17 (m, 26H), 1.16– 1.06 (m, 6H), 0.91–0.78 (m, 21H). ^13^C NMR (126 MHz, CDCl_3_) δ 173.1, 171.2, 171.0, 167.0, 163.5,132.2, 131.3, 124.5, 121.3, 114.1, 109.8, 103.89, 68.1, 63.0, 36.9, 35.3, 34.2, 31.9, 30.3, 29.7, 29.6, 29.4, 29.1, 29.0, 25.9, 25.7, 25.3, 22.7, 21.0, 21.04, 19.3, 17.6, 14.1.

## 3. Result and discussion

**Figure 1:**
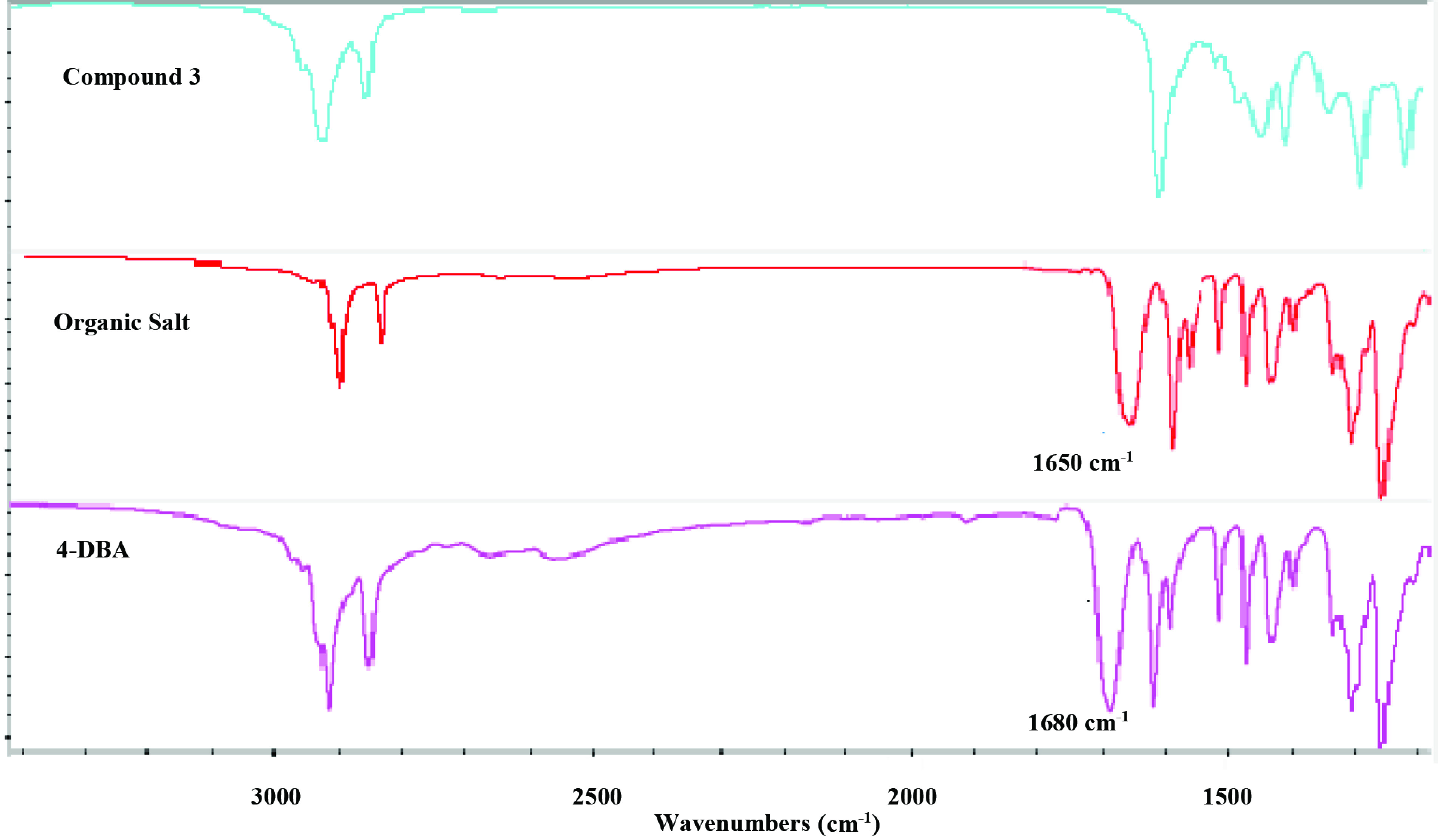
FTIR spectra of macromolecule 3, organic salt 5, and benzoic acid 4-DBA.

The formation of the salt 5 between the macromolecule 3 and the mesogenic carboxyl group was mainly studied by FTIR, H NMR, and C NMR. The comparison of FTIR spectra of macromolecule 3, free acid 4, and salt 5 are provided in Figure 1. A sharp peak belonging to the asymmetric stretching of carbonyl peak of the carboxylic acid appeared at 1680 cm^-1^ , which shifted in the salt form to 1650 cm^-1^ [26].

**Figure 2:**
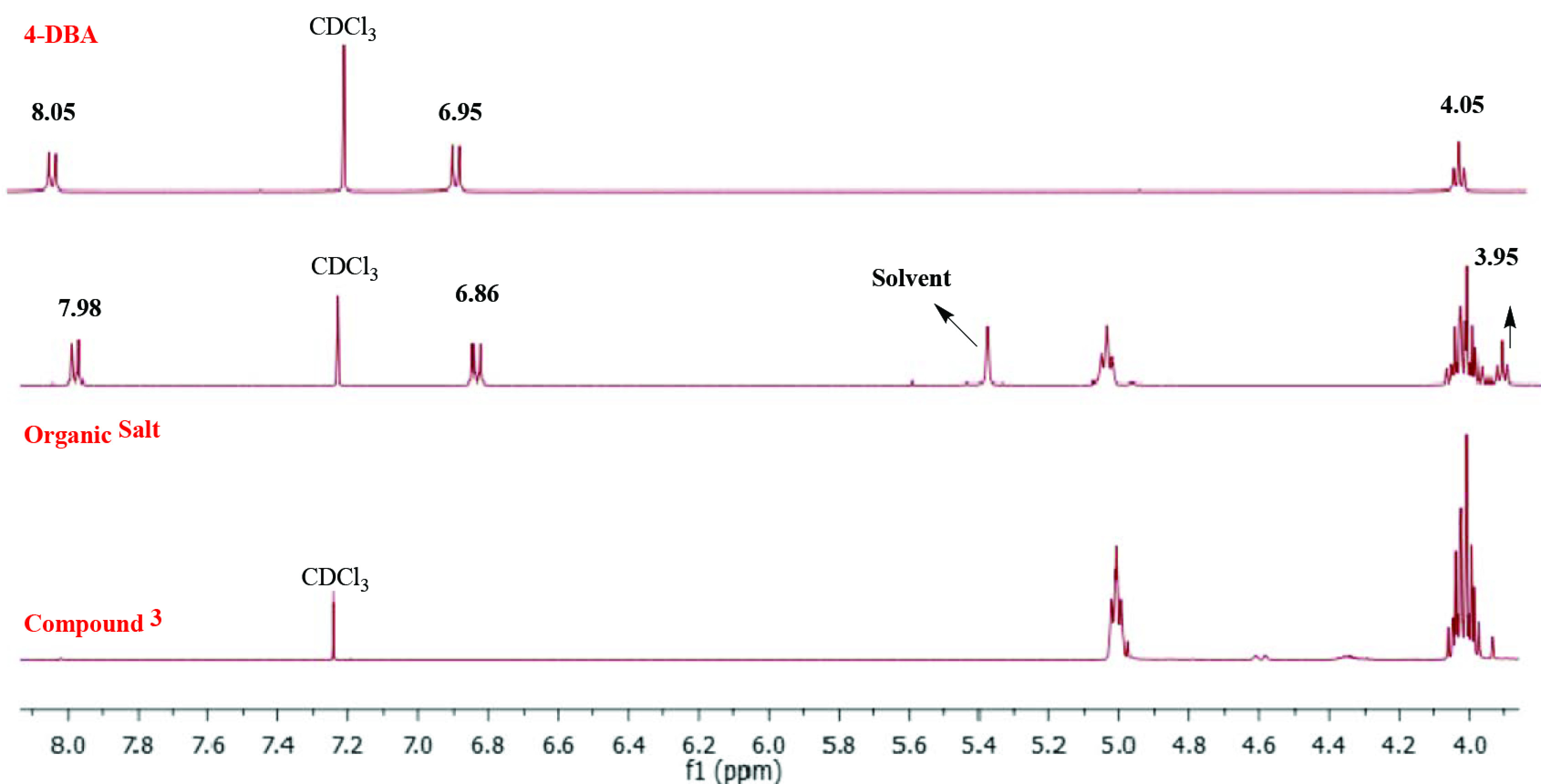
1H NMR spectra of macromolecule 3, organic salt 5, and benzoic acid 4.

The NMR spectroscopic analysis indicated the interaction between macromolecule 3 and 4-DBA. Evidences were found on the chemical shift of the aromatic hydrogens of 4-DBA shifted from 8.05 ppm and 6.95 ppm to 7.98 ppm and 6.86 ppm, indicating a change in the electron density after the formation of salt 5. Likewise, the signals of –OCH_2_ –protons of 4-DBA in the complex shifted to a higher ?eld at 3.95 ppm compared to the signals of pure 4-DBA at 4.05 ppm (Figure 2). As expected, the multiplet signals in H NMR spectra at 4.15–3.97 ppm of compound 3 showed no shifting after the complexation since their electronic environment was not influenced.

**Figure 3:**
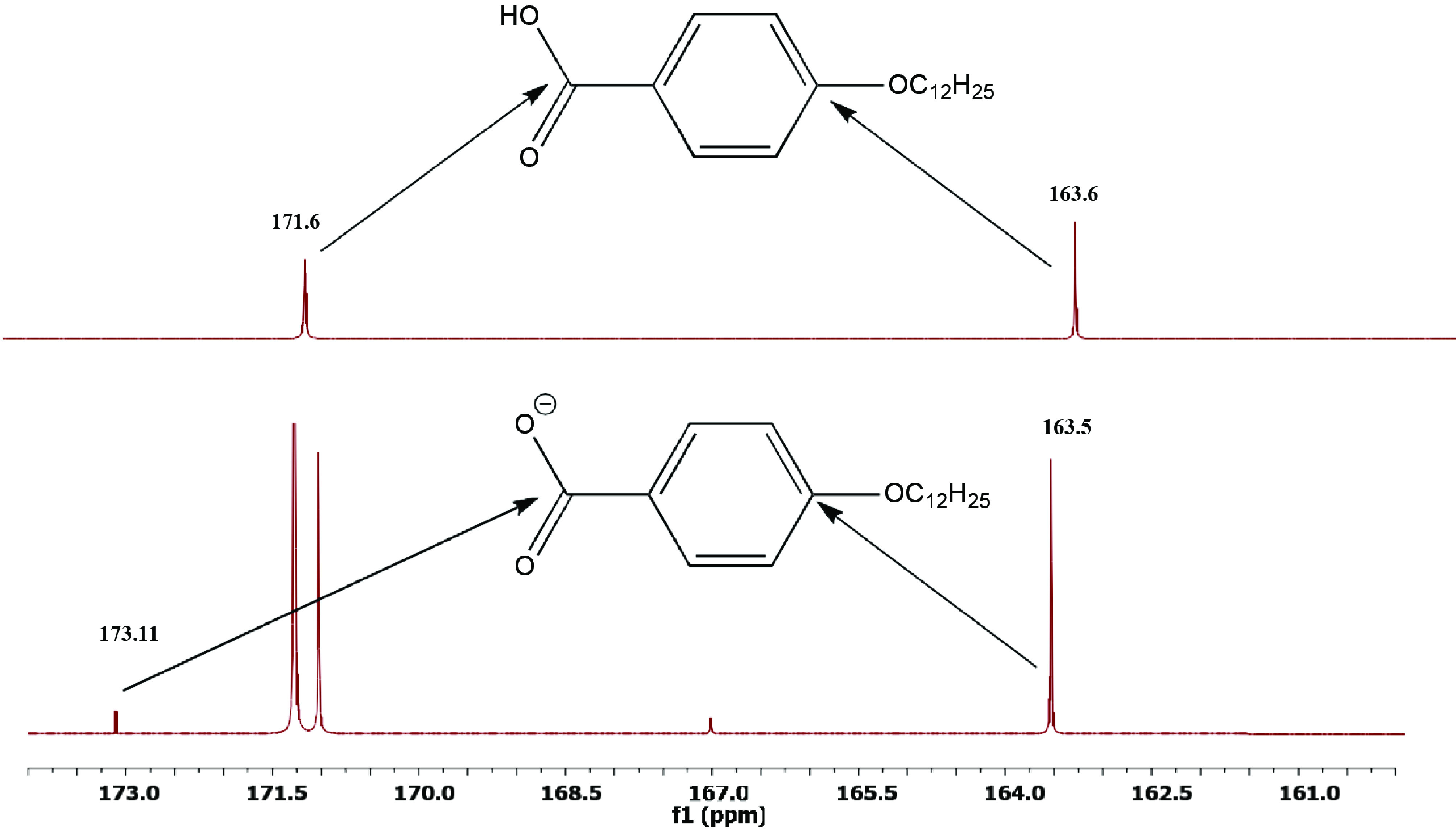
A section of 13C NMR spectra of benzoic acid 4-DBA organic salt 5.

Moreover, the ^13^C NMR spectra showed a shift of the peak of carbonyl carbon from 171.6 in compound 3 to 172.5 ppm in the salt 5, while the aromatic carbon bounded to the alkoxy group shifted slightly from 163.6 to 163.5 ppm, respectively (Figure 3).

The mesomorphic properties of the compound 4-DBA and organic salt 5 were investigated by using POM and DSC. The phase transitions of the corresponding molecules are given in Table.

**Table:** Mesophases and phase transition temperatures as observed on heating (H→) and cooling (←C) and corresponding transition enthalpies of the 4-DBA and organic salt 5. (The peak temperatures were expressed in degree Celsius and the numbers in parentheses referred to the transition enthalpy (ΔH) in kJ mol^-1^) .

Comp.	T/°C [ΔH kJ/mol]^a^
4-DBA^b^	H →: Cr 99.98 [39.01] SmC 132.43 [2.39] N 138.42 [2.05] Iso
OS(5)	H →: Cr 93.64 [98.52] SmC 115.45^c^ Iso Cr1 65.82 [19.19] Cr2 81.22 [27.03] SmC 102.5 [5.1] Iso: ← C

^a^Perkin-Elmer DSC-6; enthalpy values in italics in brackets taken from the 2nd heating and cooling scans at a rate of 10 °C min^-1^; abbreviations: Cr = crystalline, SmC= tilted smectic phase, N= nematic phase; Iso = isotropic liquid phase. ^b^[14,15] Cr 95.1 SmC 128.9 N 137.2 Iso [16] Cr 92.4 SmC 131.5 N 142.0 Iso ^c^ confirmed by POM.

**Figure 4:**
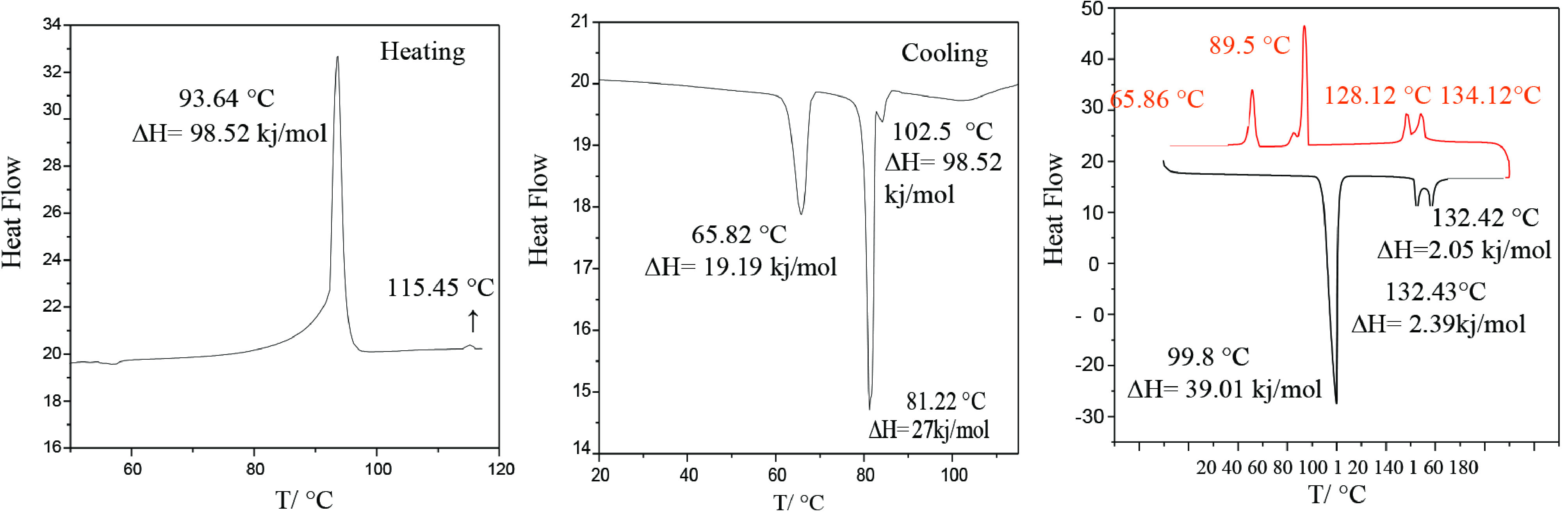
DSC thermograms of heating (left), cooling (centre) organic salt, and 4-DBA (right) on 2nd heating and cooling (10 °C min-1).

**Figure 5:**
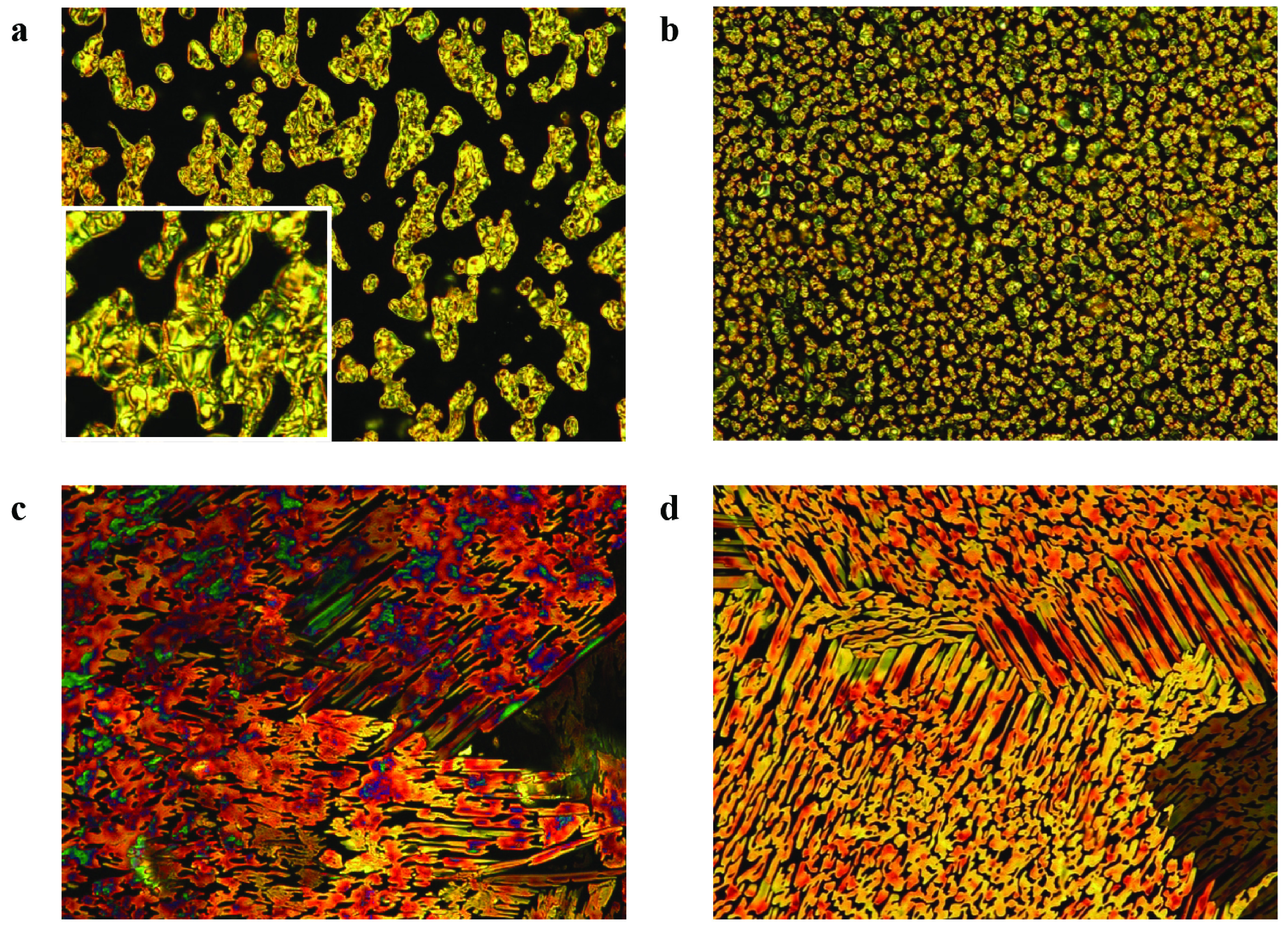
Optical textures of organic salt 5 as observed between crossed polarizers in an ordinary glass plate; (a) texture of SmC phase at 94 °C heating and (b) 89 °C cooling (c) texture of crystal phase at 77 °C, d) texture of crystal phase at 74 °C (magnification ×100).

As shown in Figure 4, compound 4-DBA with n-dodecyloxy alkyl chain showed enantiotropic liquid crystalline properties that were in agreement with the behaviour observed for the analogous benzoic acidscarrying an alkoxy chain with different numbers of carbon atoms at the 4-position of the aromatic ring [21-27,29]. The organic salt 5 exhibited a phase transition sequence of Cr1 -Cr2 –SmC-Iso which was in agreement with 3 endotherms in DSC cooling curves (Figure 4 centre). Upon heating from the crystal, the SmC texture was observed at 93–115 °C by POM (Figure 5). The texture composed of a disc-like shaped π-conjugated system based on 1,3,5-triazine central core and the mesogenic carboxyl group referred to here as the Smectic C phase [30-33].

Figure 5a shows Schlieren texture of SmC mesophase. The inset in clearly shows Schlieren regions of the texture.

The creation of SmC mesophase is explained by the ionic interaction between the triarmed π-conjugated system and the mesogenic carboxyl group. In this noncovalent intermolecular interaction, the star-shaped compound 3 acts as proton acceptor and the benzoic acid with n-dodecyloxy group acts as the proton donor. The ionic interactions between macromolecule 3 and 4-DBA have striking influence on the mesogenic properties such as melting and clearing temperatures as well as prompting SmC phase at lower temperatures.

## 4. Conclusion

A new triazine-based macromolecular π-conjugated system carrying chiral citronellyloxy chains positioned at the peripheries was synthesized via Sonogashira and Negishi cross coupling reactions. Triazines at the periphery bearing citronellyloxy groups were connected to the central triazine unit by 3 acetylenic bridges. Its organic salt was also prepared, which was resulted from hydrogen bonding interaction between macromolecule 3 and 4-DBA mixed with 1:1 ratio in THF. The resulting organic salt was characterized by ^1^H NMR, ^13^C NMR, and FTIR. The organic salt exhibited enantiotopic SmC phase texture at lower temperatures.

The presence of chiral citronellyloxy groups on the periphery lowered the mesophase transitions from SmC 132.43 °C for linear alkyl chain substituted analogue structure [13] to SmC 115.45 °C in the organic salt 5. Low temperature mesophase transitions are desirable for low temperature applications of liquid crystals.

Supplementary Materials
