# Supplementary material for: Synthesis and liquid crystalline properties of new triazine-based π-conjugated macromolecules with chiral side groups
Source: Turk J Chem. 2020 Jun 1;44(3):726–35. doi: 10.3906/kim-1912-51 (PMC7671215; doi:10.3906/kim-1912-51)
Supplement: Supplementary file 1 — Supplementary Materials [file turkjchem-44-726-sup001.pdf]

Synthesis and liquid crystalline properties of new triazine-based π-conjugated macromolecules with chiral side groups

Nihat AKKURT,[1,2] Mohammed Hadi Ali AL-JUMAILI,[1] Hale OCAK,[1] Fatih ÇAKAR,[1] Lokman TORUN[1,3]

Supporting Information

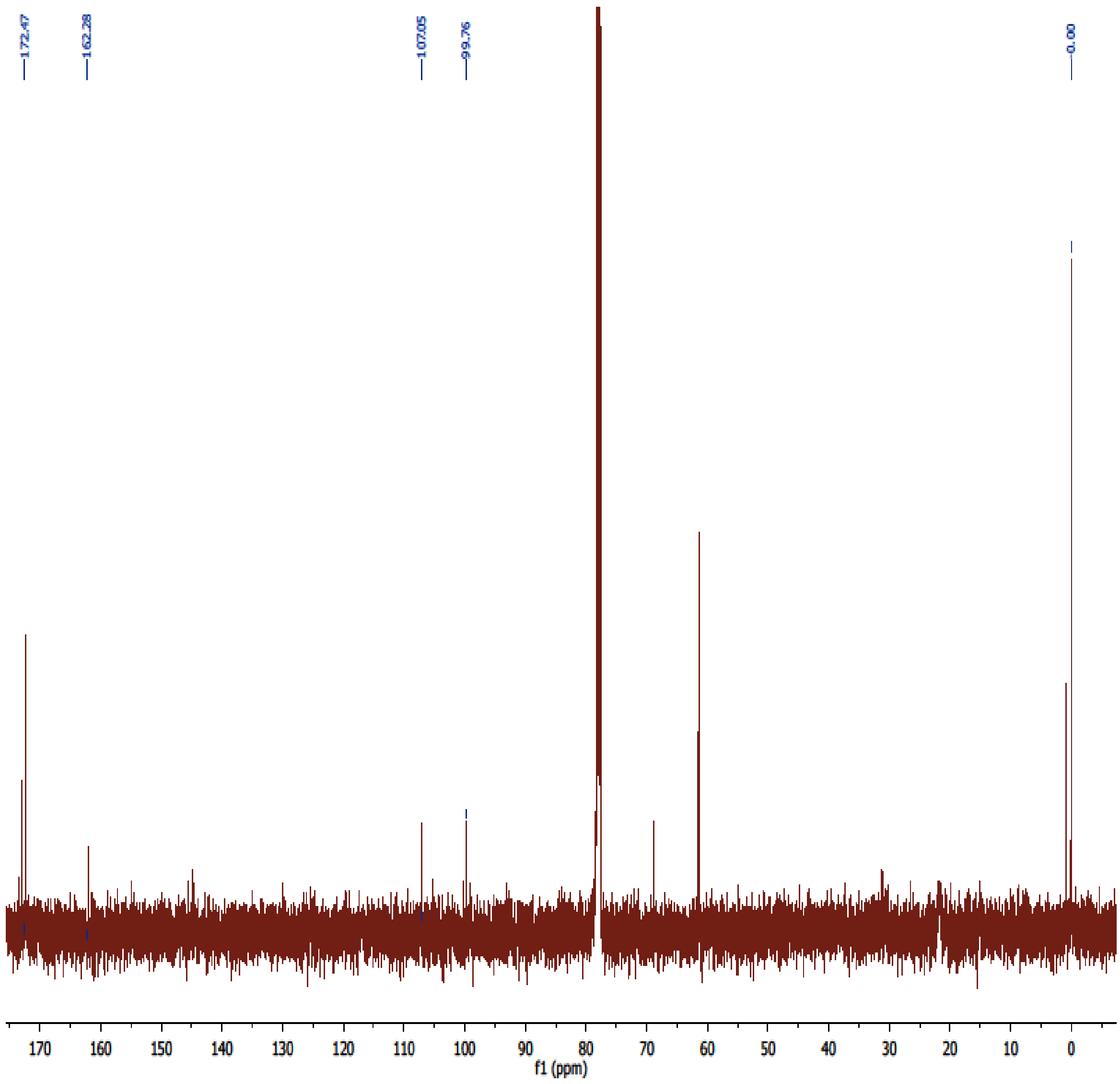

Figure 6. ^{13}C NMR result of compound **1**.

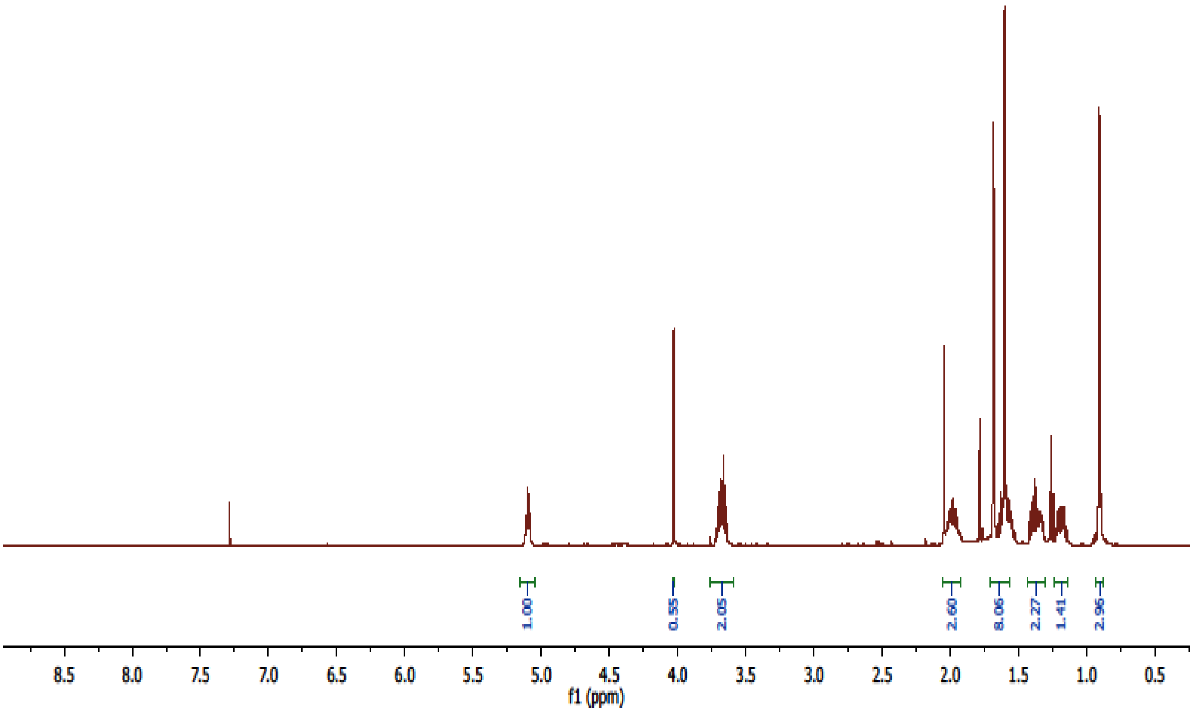

Figure 7. ^{1}H NMR result of compound **2**.

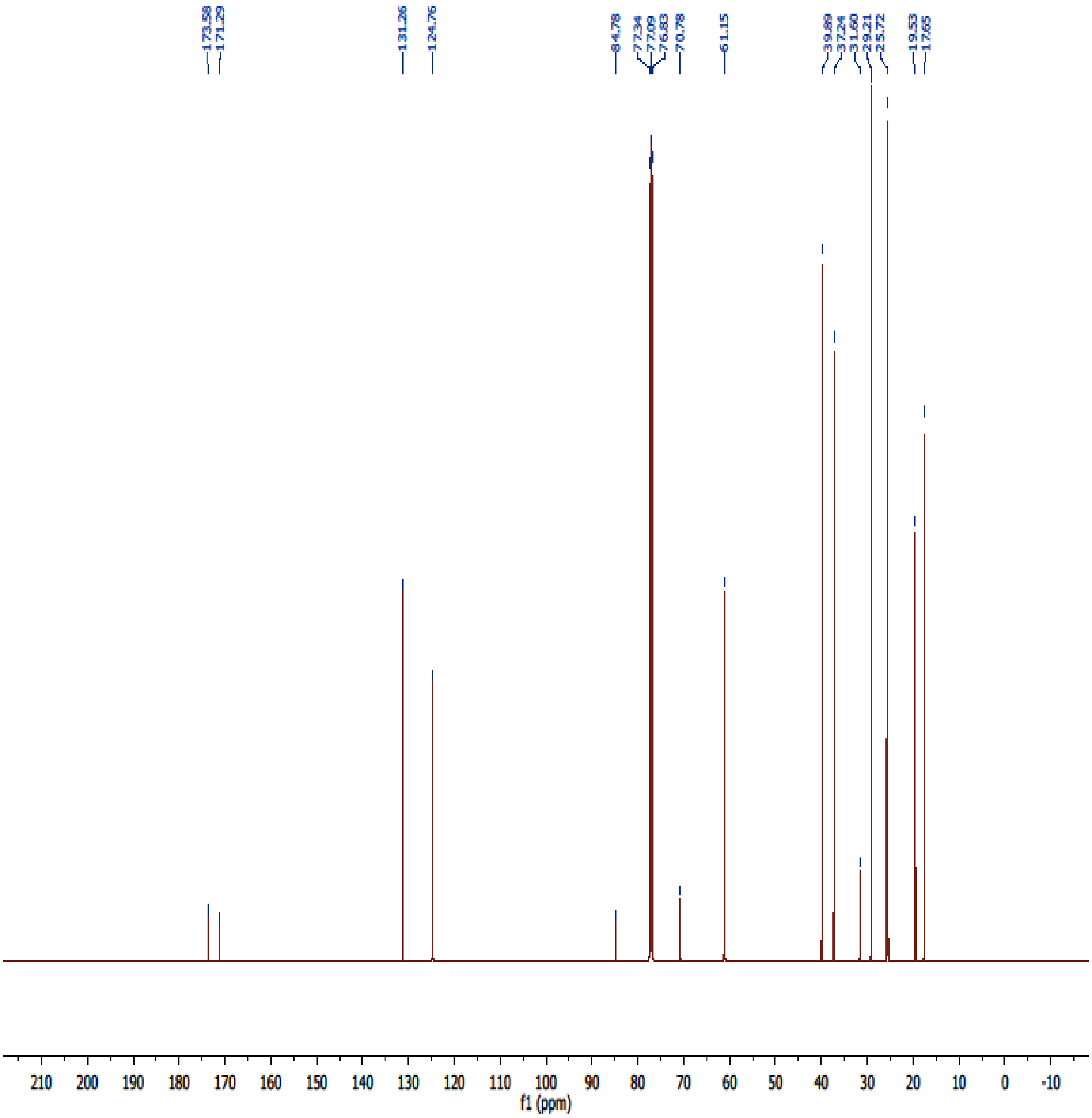

Figure 8. ^{13}C NMR result of compound **2.**

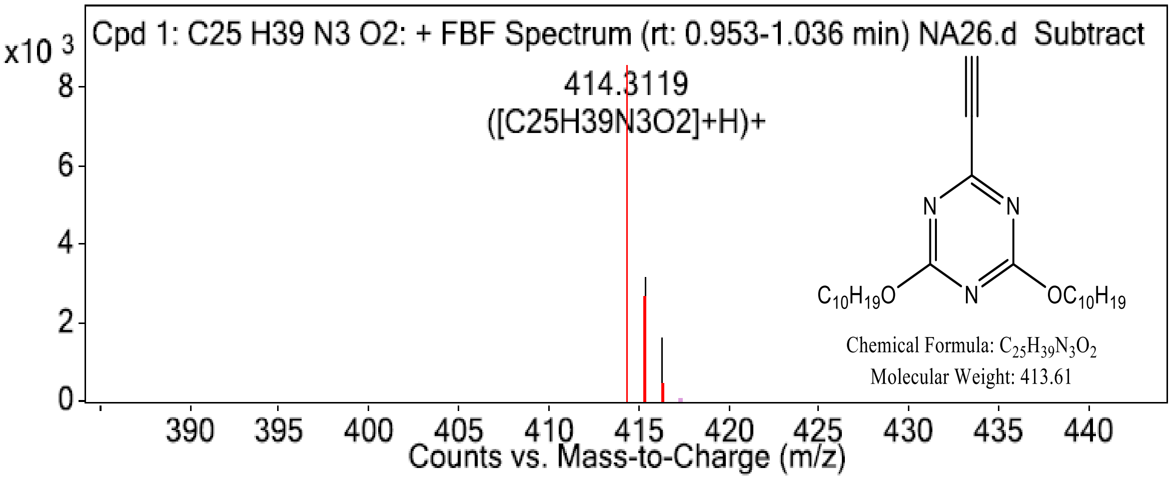

MS Spectrum Peak List

m/z	z	Abund	Formula	Ion
414.3119	1	7570.73	C25H39N3O2	(M+H)+
415.3135	1	3146.02	C25H39N3O2	(M+H)+
416.3179	1	1587.85	C25H39N3O2	(M+H)+

Figure 9. Q-TOF result of compound **2**.

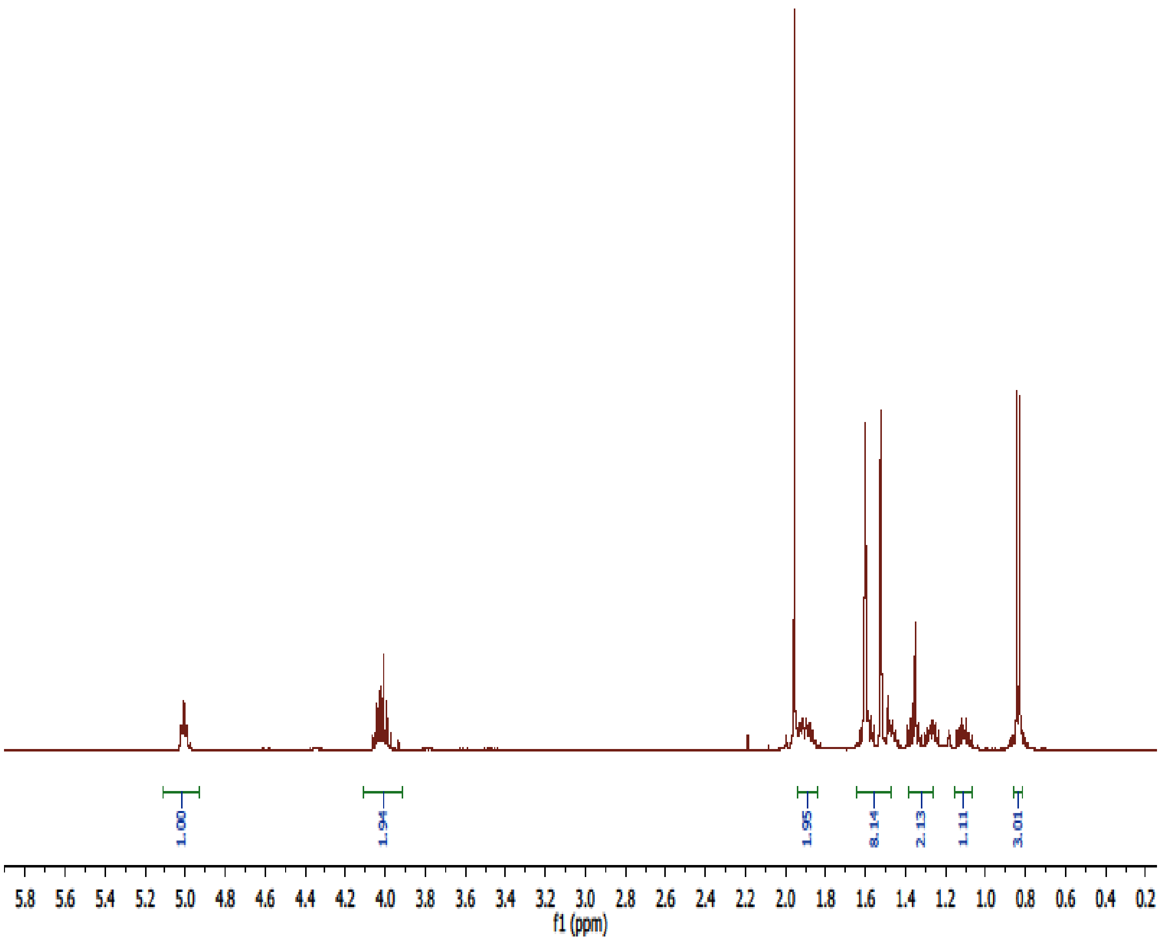

Figure 10. ^{1}H NMR result of compound **3**.

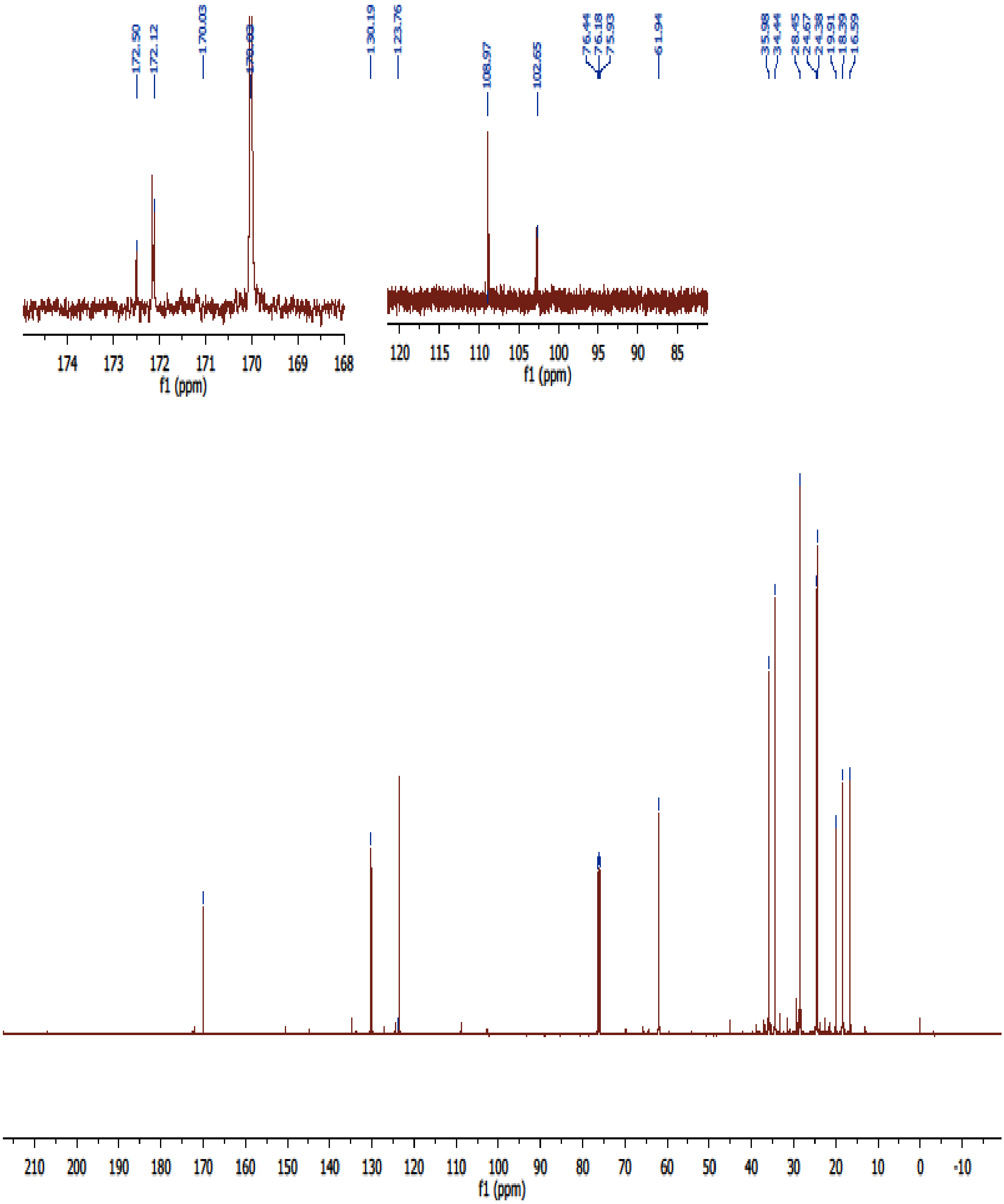

Figure 11. ^{13}C NMR result of compound **3**.

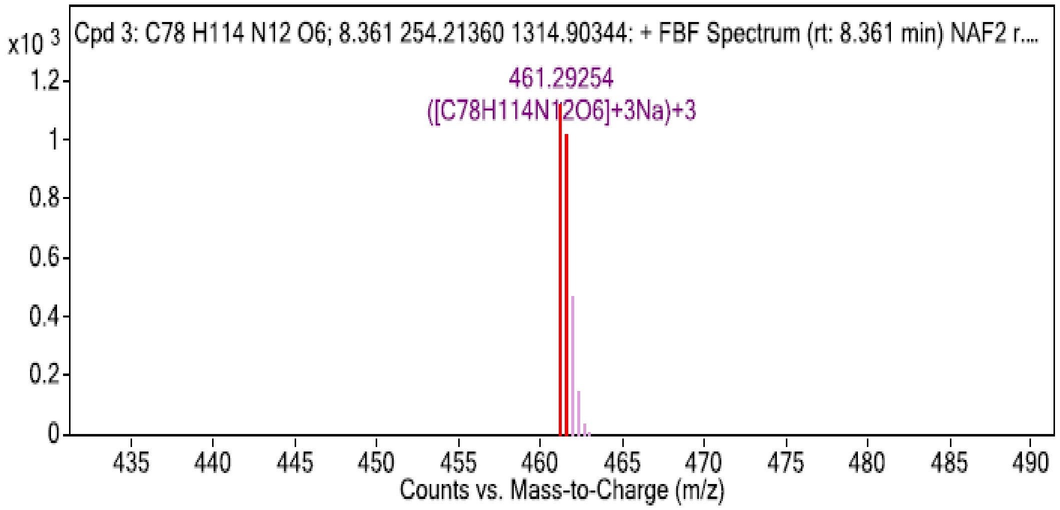

MS Spectrum Peak List

m/z	z	Abund	Formula	Ion
461.29254	3	1125	C78H114N12O6	(M+3Na)+3
461.61682	3	309.1	C78H114N12O6	(M+3Na)+3

Figure 12. Q-TOF result of compound **3**.

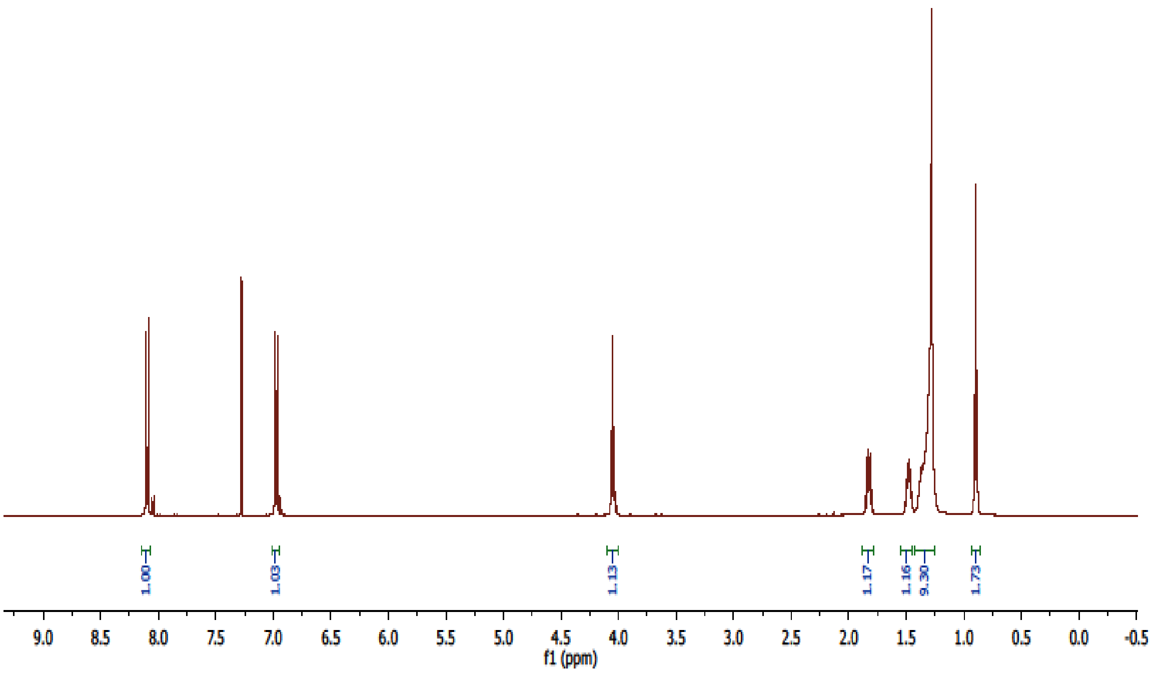

Figure 13. ^{1}H NMR result of compound **4**.

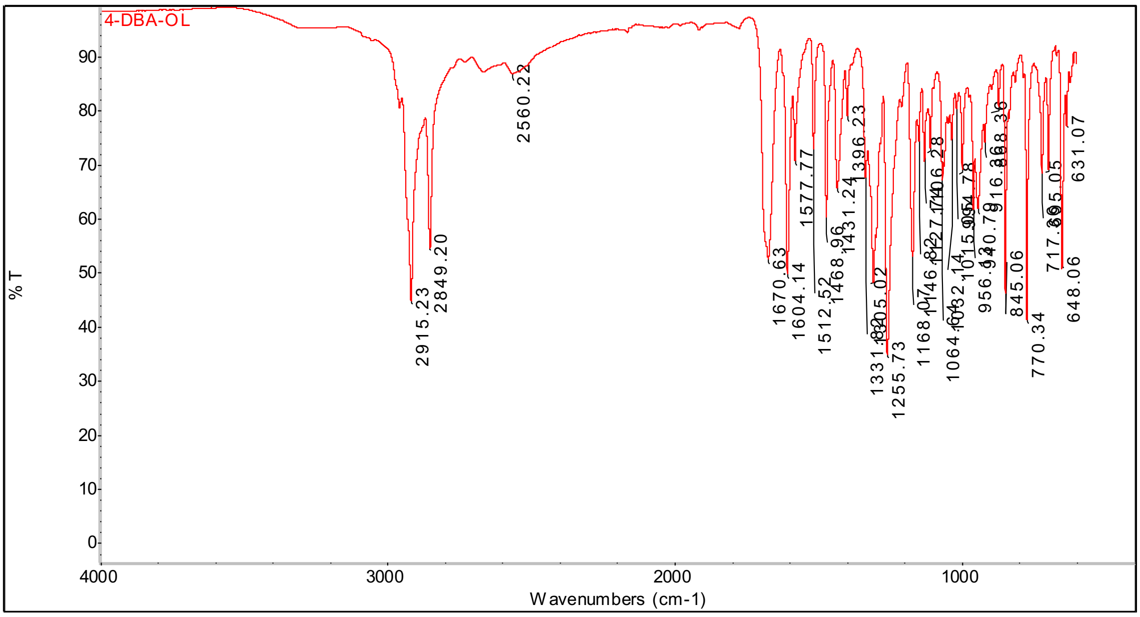

Figure 14. FTIR result of 4-DBA.

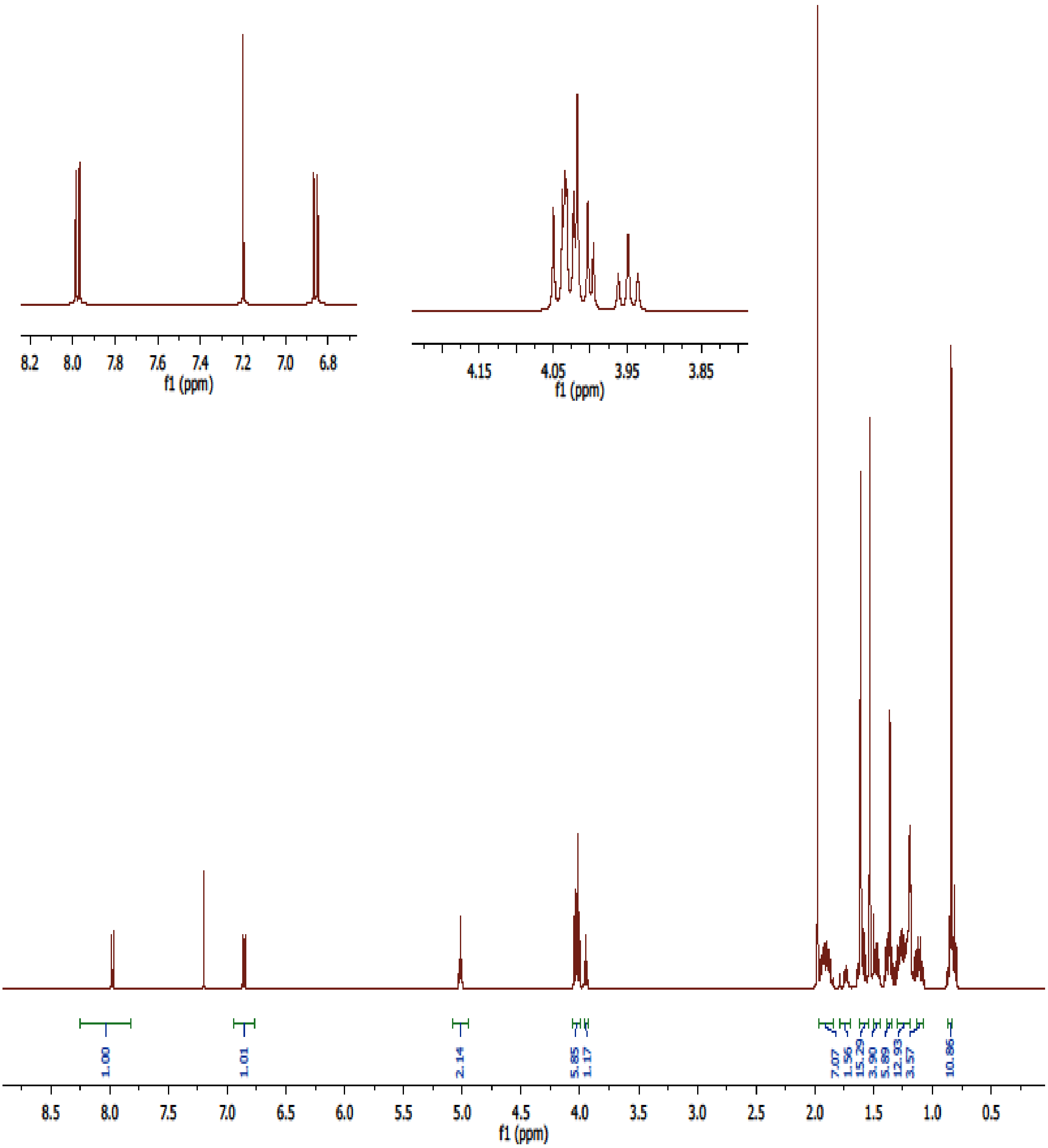

Figure 15. ^{1}H NMR result of organic salt (**5**).

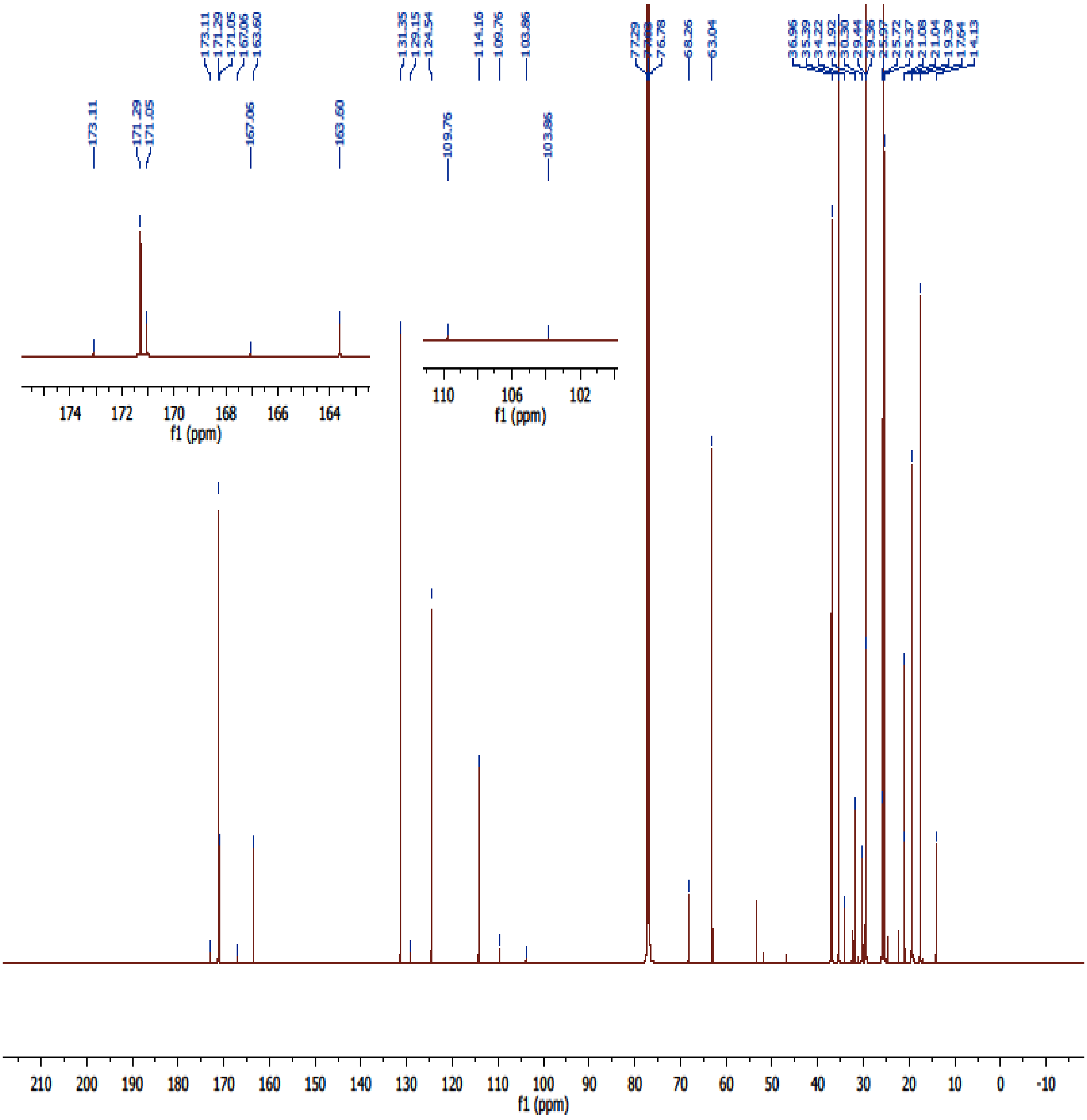

Figure 16. ^{13}C NMR result of organic salt (**5**).
